# Early Versus Delayed Oral Anticoagulation in Patients With Acute Ischemic Stroke Due to Atrial Fibrillation: A Meta-Analysis

**DOI:** 10.7759/cureus.40801

**Published:** 2023-06-22

**Authors:** Gayathri Tirumandyam, Gautham Varun Krishna Mohan, Lokeshwar Raaju Addi Palle, Ibrahim Reyaz, Salar Haider, Madiha D Haseeb, Faraz Saleem

**Affiliations:** 1 Internal Medicine, Siddhartha Medical College, Dr Nandamuri Taraka Rama Rao (NTR) University of Health Sciences, Tirupathi, IND; 2 Internal Medicine, Tirunelveli Medical College, Tirunelveli, IND; 3 Department of Surgery, Kamala Children's Hospital, Chennai, IND; 4 Internal Medicine, Christian Medical College and Hospital, Ludhiana, IND; 5 Medicine, King Edwards Medical University, Islamabad, PAK; 6 Neurology, Dow University of Health Sciences, Karachi, PAK; 7 Internal Medicine, California Institute of Behavioral Neurosciences and Psychology, Fairfield, USA; 8 Internal Medicine, Akhtar Saeed Medical and Dental College, Lahore, PAK

**Keywords:** ischemic stroke, atrial fibrillation, delayed, early, oral anticoagulants

## Abstract

The aim of this study was to compare the safety and efficacy of early oral anticoagulation with delayed anticoagulant therapy in patients who have had a recent stroke and have atrial fibrillation (AF). This meta-analysis was conducted following the Preferred Reporting Items for Systemic Reviews and Meta-analyses (PRISMA) statement. The literature search was independently performed by two authors. We searched PubMed and Scopus using search strings that included the following terms: "stroke," "atrial fibrillation," "oral anticoagulants," "recurrent stroke," and "intracerebral hemorrhage." Our search spanned from the inception of databases to May 25, 2023. The primary outcome assessed in this study was the composite efficacy outcome (as defined by individual studies). Recurrent ischemic stroke (IS), intracranial hemorrhage (ICH), and death from any cause were assessed as secondary outcomes. For safety analysis, bleeding events were compared between the two study groups. We included five articles in this meta-analysis, comprising a total of 7958 patients (including 3793 in the early treatment group and 4165 in the delayed treatment group). Pooled analysis showed that the risk of composite efficacy outcome (RR: 0.69, 95% CI: 0.51-0.93, p-value: 0.01) and recurrent ischemic stroke (RR: 0.71, 95% CI: 0.53-0.94, p-value: 0.02) were lower in the early treatment group. However, no significant differences were observed between the two groups in terms of all-cause mortality, intracranial hemorrhage, or bleeding events. In light of the findings, healthcare professionals should carefully evaluate the risks and benefits of early versus delayed DOAC treatment in individual patients, considering factors such as stroke severity, bleeding risk, and patient preferences.

## Introduction and background

Atrial fibrillation (AF) is linked to a significantly higher risk of stroke, up to five times greater than the average [1]. Compared to other causes of cardioembolic strokes, AF-related strokes are more likely to result in adverse functional outcomes at three months [2]. Additionally, AF is associated with an increased likelihood of the early recurrence of ischemic strokes (ISs) [3]. Oral anticoagulant therapy (OAC) can decrease the risk of systemic embolism and ischemic stroke among individuals with AF [4], but the optimal timing of OAC after an acute ischemic stroke (AIS) or transient ischemic attack (TIA) is unknown [5]. Preventing early recurrence is a critical clinical challenge in cases of acute ischemic stroke associated with AF. The risk of recurrence within 7-14 days in these cases ranges from 0.4% to 1.3% per day [6-7]. AF-related ischemic strokes are more likely to result in disability or death compared to other types of strokes. They are associated with longer hospital stays and higher costs [8]. Therefore, it is crucial to address this risk and prevent early recurrence to improve patient outcomes.

The timing of initiating OAC after a stroke is a complex decision. Early initiation within the first few days after a stroke could potentially prevent the recurrence of ischemic strokes. However, it carries the risk of symptomatic intracranial hemorrhage (ICH), including the hemorrhagic transformation of the infarct. The estimated risk of such complications, particularly in the first seven days, is approximately 9% [9]. Consequently, there is uncertainty among clinicians regarding the optimal timing for starting OAC. Recent observational studies, primarily involving patients treated with warfarin or other vitamin K antagonists, have reported an 8% to 10% risk of recurrent ischemic stroke within 90 days following an AF-related ischemic stroke. Additionally, these studies have noted a 2% to 4% risk of symptomatic intracranial hemorrhage during the same period [10-11].

Given that direct oral anticoagulants (DOACs) have demonstrated comparable effectiveness to vitamin-K antagonists (VKAs) in preventing IS in individuals with AF while being safer in relation to ICH, major bleeding, and overall mortality [12-13], it is reasonable to question whether there are any differences in terms of their safety and efficacy when given during the early phase after an ischemic stroke (within 14 days).

Considering the lack of high-quality studies, recommendations related to the timing of initiation of anticoagulation have varied. Therefore, we sought to use past studies to compare the safety and efficacy of early oral anticoagulation with delayed anticoagulant therapy in patients who have had a recent stroke and have atrial fibrillation.

## Review

Methodology

This meta-analysis was conducted following the Preferred Reporting Items for Systemic Reviews and Meta-analyses (PRISMA) statement.

Search Strategy and Study Selection

The literature search was independently performed by two authors. We searched PubMed and Scopus using search strings that included the following terms: "stroke," "atrial fibrillation," "oral anticoagulants," "recurrent stroke," and "intracerebral hemorrhage." Our search spanned from the inception of databases to May 25, 2023. No restrictions were placed on the language or year of publication. Additionally, we manually searched the reference lists of all included articles to ensure the inclusion of all relevant studies.

We included randomized controlled trials (RCTs) and observational cohort studies that presented patients administered oral anticoagulants after atrial fibrillation-related ischemic stroke. We excluded case series, case reports, editorials, and narrative reviews. Studies lacking a comparison group were also excluded. All eligible studies were independently reviewed by two authors. The first-level screening was done using abstracts and titles, followed by full-text screening. Any disagreements in the process of the search strategy and study selection were resolved through discussion.

Data Extraction, Study Endpoints, and Quality Assessment

Data from the included studies were extracted using a pre-designed data extraction form in Microsoft Excel. The extracted data included author names, year of publication, study design, study groups, sample size, follow-up duration, and baseline characteristics. The primary outcome assessed in this study was the composite efficacy outcome (as defined by individual studies). Recurrent ischemic stroke, intracranial hemorrhage, and death from any cause were assessed as secondary outcomes. For safety analysis, bleeding events were compared between the two study groups. The quality assessment of individual studies was independently performed by two authors using the Newcastle-Ottawa Scale for cohort studies and the Cochrane Risk of Bias Assessment for the quality assessment of RCTs. Data extraction and quality assessment were conducted independently by two authors, and any disagreements in these steps were resolved through discussion.

Statistical Analysis

For dichotomous variables, we calculated the corresponding risk ratios (RR) and 95% confidence intervals (95% CI) for the comparison of early and late OAC treatment. A p-value of <0.05 was considered significant. To qualitatively assess heterogeneity, I2 values above 50% were considered indicative of substantial heterogeneity, while values exceeding 75% were regarded as reflecting considerable heterogeneity. The significance level for the Q statistic was set at 0.1. In cases of significant heterogeneity, a random-effects model was used; otherwise, a fixed-effects model was used. All statistical analyses were performed using the Cochrane Collaboration's Review Manager (RevMan 5.4.1) Software Package (Copenhagen: The Nordic Cochrane Centre, The Cochrane Collaboration, 2014).

Results

**Figure 1:**
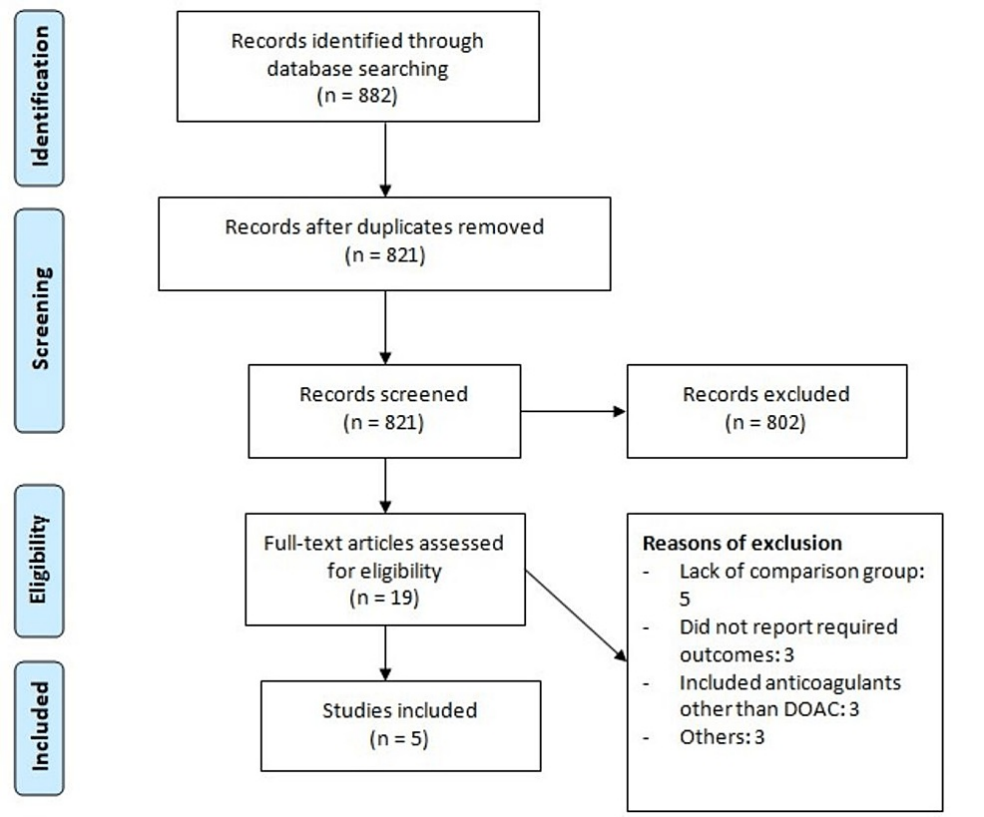
PRISMA flowchart of study selection

The systematic database search yielded 882 articles. After removing duplicates, 821 studies were initially screened using abstracts and titles, and 28 studies were potentially eligible for inclusion. The full text of 19 studies was obtained, and a detailed assessment was done to assess whether they were eligible or not. After assessing full-text records, we identified five articles to be included in this meta-analysis, comprising a total of 7958 patients (including 3793 in the early treatment group and 4165 in the delayed treatment group). Figure 1 shows the study selection process. Table 1 shows the characteristics of the included studies. Out of five studies, two were RCTs and three were observational studies. Table 2 presents the quality assessment of all included studies.

**Table 1:** Characteristics of included studies RCT: randomized-control trial; NIHSS: National Institutes of Health (NIH) Stroke Scale

Author	Year	Study Design	Groups	Duration	Sample Size	Follow-up (years)	Age (years)	Mean NIHSS
Fischer et al. [14]	2023	RCT	Early	Minor/moderate stroke: 48 hour; major stroke: day 6 or 7	1006	90 days	77.5	3
			Late	Minor stroke: day 3 or 4; moderate stroke: day 6 or 7; major stroke: day 12, 13 or 14	1007			
Marchis et al. [15]	2021	Observational	Early	≤4 days	1362	30 days	77.5	5
			Late	≤5 days	1188			
Oldgren et al. [16]	2022	RCT	Early	≤4 days	450	90 days	78.3	6.1
			Late	5 to 10 days	438			
Wilson et al. [17]	2018	Observational	Early	≤4 days	358	90 days	76	4
			Late	≥5 days	997			
Yaghi et al. [18]	2020	Observational	Early	≤3 days	617	90 days	77.5	7.5
			Late	≥ days	535			

**Table 2:** Quality assessment

Quality assessment for observational studies					
Study ID	Selection	Comparability	Outcome		
Marchis et al. [15]	3	2	3		
Wilson et al. [17]	4	1	2		
Yaghi et al. [18]	4	2	3		
Quality assessment for randomized control trial					
Study ID	Selection	Performance	Attrition	Reporting	Other
Fischer et al. [14]	No	Low	Low	Low	Low
Oldgren et al. [16]	No	Low	Low	Unclear	Low

Meta-Analysis of Outcomes

**Figure 2:**
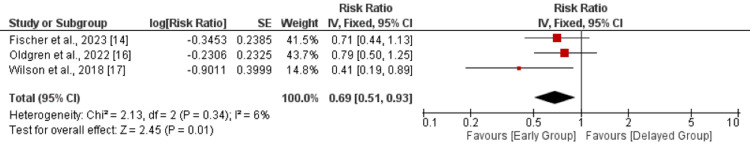
Composite efficacy outcomes Sources: References [14,16,17]

Composite efficacy outcome: Three studies were included in the pooled analysis of composite efficacy outcomes. As shown in Figure 2, risk of composite outcome was significantly lower in patients receiving OAC early compared to its counterparts (RR: 0.69, 95% CI: 0.51-0.93, p-value: 0.01). No significant heterogeneity was reported among the study results (I-square: 6%, p-value: 0.34).

**Figure 3:**
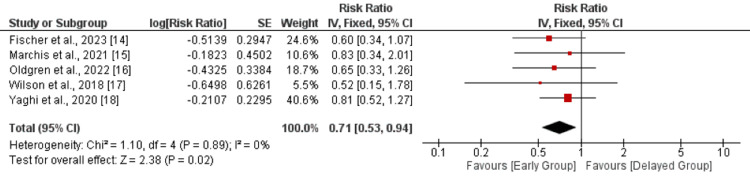
Recurrent ischemic stroke Sources: References [14-18]

Recurrent ischemic stroke: Five studies were included in the comparison of recurrent ischemic stroke between the early and delayed DOAC treatment groups. As shown in Figure 3, a pooled analysis showed that the risk of recurrent ischemic stroke was 29% lower in the early treatment group compared to the delayed treatment group (RR: 0.71, 95% CI: 0.53-0.94, p-value: 0.02). No significant heterogeneity was reported in the study (I-square: 0%, p-value: 0.89).

**Figure 4:**
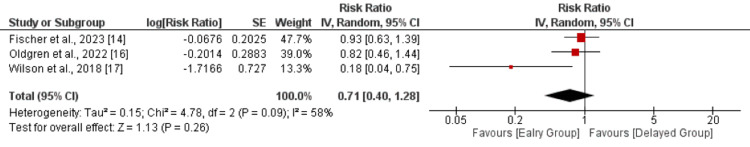
All-cause mortality Sources: References [14,16-17]

All-cause mortality: Three studies compared all-cause mortality between the early and delayed DOAC treatment groups. As shown in Figure 4, no significant differences were reported between the two groups in terms of the risk of all-cause mortality (RR: 0.71, 95% CI: 0.40-1.28, p-value: 0.26). Significant heterogeneity was reported among the study results (I-square: 58%, p-value: 0.09). All three studies reported low mortality in the early treatment group. However, only one study showed a significant difference.

**Figure 5:**
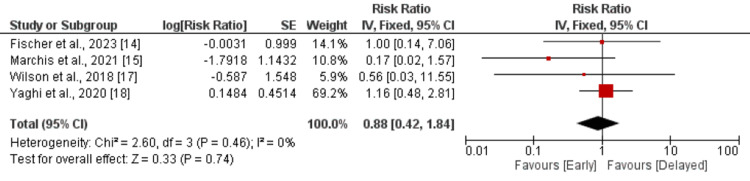
Intracranial haemorrhage Sources: References [14,15,17,18]

Intracranial hemorrhage: Four studies compared the incidence of intracranial hemorrhage between the early and delayed DOAC treatment groups. Pooled analysis showed that the risk of intracranial hemorrhage was lower in patients in the early treatment group, but the difference is statistically insignificant (RR: 0.88, 95% CI: 0.42-1.84, p-value: 0.74), as shown in Figure 5. No significant heterogeneity was reported in the study (I-square: 0%, p-value: 0.46). The study conducted by Oldgren et al. did not report any intracranial hemorrhage events in any group.

**Figure 6:**
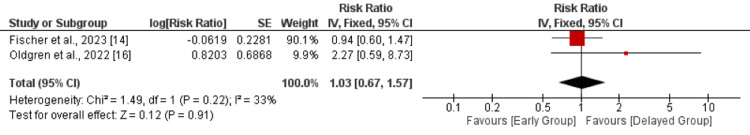
All-bleeding events Sources: References [14,16]

Bleeding events: A pooled analysis of two studies comparing the risk of bleeding events between two groups showed that no significant difference was reported between the two groups in terms of the risk of bleeding events (RR: 1.03, 95% CI: 0.67-1.57, p-value: 0.91), as shown in Figure 6. No significant heterogeneity was reported in the study (I-square: 33%, p-value: 0.22).

Discussion

In the present meta-analysis, which included data from xxx ischemic stroke patients with AF, we found a higher risk of recurrent ischemic stroke in patients who received early DOAC treatment compared to those who received delayed DOAC treatment. However, no significant differences were observed between the two groups in terms of all-cause mortality, intracranial hemorrhage, or bleeding events.

Previous studies investigating the timing of initiating OACs after AIS have yielded conflicting results. Paciaroni et al. discovered that the most favorable period to begin OACs was between 4 and 14 days following the onset of stroke [10]. These results align with those from the RAF-NOAC study, which observed that individuals who initiated non-vitamin K antagonist oral anticoagulants (NOACs) between 3 and 14 days had the lowest combined rates of recurrent stroke and significant bleeding [19]. In 2018, the American Heart Association/American Stroke Association guidelines recommended initiating OACs for secondary prevention within the first two weeks [20]. Conversely, guidelines in the United Kingdom advised delaying OAC administration until at least 14 days after the onset of a disabling ischemic stroke [21]. However, more recent studies have contradicted the recommendation of waiting 14 days before initiating anticoagulation treatment. Yaghi et al. conducted a registry study involving eight comprehensive stroke centers, and their findings did not support an increased risk of recurrent ischemic events or intracranial hemorrhage when OACs were initiated within the zero- to three-day period compared to initiation between 4 and 14 days [18]. Given that delayed initiation of OACs did not demonstrate clear clinical benefits, early use of OACs in acute ischemic stroke patients with AF may be a reasonable alternative. Our meta-analysis findings are consistent with the results of the two recent RCTs [22-23], which provided reassurance regarding the safety of early initiation of OACs in patients with mild to moderate ischemic stroke.

Previous guidelines suggesting a delay in initiating OACs after acute ischemic stroke were primarily driven by concerns regarding hemorrhagic transformation. However, our analysis indicates that early use of OACs for secondary prevention in patients with AF and ischemic stroke has benefits in terms of preventing recurrent ischemic stroke [24].

In current clinical practice, it is a common approach of delaying the start of anticoagulation treatment following an ischemic stroke. This approach is recommended by various guidelines that are based on expert consensus. For example, European guidelines suggest evaluating the severity of the stroke using the NIHSS score and waiting three days after a minor stroke, six days after a moderate stroke, and 12 days after a severe stroke before initiating anticoagulation, as determined by this score. The guidelines from the American Stroke Association [25] also recommend delaying anticoagulation for more than 14 days if there is a high risk of the ischemic brain infarct developing hemorrhagic transformation. Conversely, if the risk of this complication is low, the guidelines suggest starting anticoagulation between days 2 and 14 after the stroke.

We must acknowledge certain methodological limitations in the current meta-analysis. First, the observational study designs employed in the included studies may have introduced substantial selection bias, which cannot be adequately addressed through a meta-analytical approach. Second, only two RCTs were included. Additionally, due to a lack of patient-level data, we were not able to perform subgroup analysis. Therefore, in the future, large-scale trials are needed to determine the optimum time period during which oral anticoagulant therapy can be initiated in patients with stroke and atrial fibrillation.

## Conclusions

In conclusion, our meta-analysis, which included data from 7958 ischemic stroke patients with AF, revealed that early OAC treatment was associated with a higher risk of recurrent ischemic stroke compared to delayed OAC treatment. However, no significant differences were observed between the two groups in terms of all-cause mortality, intracranial hemorrhage, or bleeding events. In light of the findings, healthcare professionals should carefully evaluate the risks and benefits of early versus delayed DOAC treatment in individual patients, considering factors such as stroke severity, bleeding risk, and patient preferences. Future research should aim to clarify the optimal time period for initiating oral anticoagulant therapy in this patient population to inform evidence-based guidelines and improve clinical decision-making.
